# Versatile use of vacuum-assisted healing in fifty patients

**DOI:** 10.4103/0970-0358.59273

**Published:** 2009

**Authors:** Ahmad Al Fadhli, George Alexander, James Roy Kanjoor

**Affiliations:** Al-Babtain Center for Plastic Surgery and Burns, IBN Sina Hospital, Post Box 25427, Safat 13115, Kuwait; 1 Belhoul Specialty Hospital, Dubai, UAE

**Keywords:** Vacuum-assisted healing vaccum assisted closure, wound healing, VAC therapy

## Abstract

**Context:**

Wound management can often be a challenging experience, especially in the presence of diabetes mellitus, vascular or immunological compromise. While no single technique can be considered by itself to be ideal, vacuum-assisted healing, which is a recent innovation, is fast becoming a necessary addition as adjuvant therapy to hasten wound healing.

**Aims:**

To determine the efficacy of vacuum-assisted healing.

**Settings and Design:**

Plastic surgery centre. Ministry of Health Hospital, Kuwait.

**Materials and Methods:**

Patients from Kuwait in a wide variety of clinical situations were chosen for study: Patients (n=50) were classified by diagnosis: Group 1: pressure sore- sacral (n= 3), trochanteric (n=6), ischial (n= 2); Group 2: ulcers (n= 11); Group 3: traumatic soft tissue wounds (n =15); Group 4: extensive tissue loss from the abdominal wall perineum, thigh and axilla (n =5); Group 5: sternal dehiscence wounds (n =4) and Group 6: wounds from flap necrosis (n =4). All wounds were subjected to vacuum by wall unit or portable unit, using pressure of 100-125 mm - continuous or intermittent. Closure of wounds, significant reduction in size and refusal by patient for continuation of vacuum-assisted closure therapy were end points of vacuum application.

**Results:**

Sixteen per cent of patients showed complete healing of the wound. Seventy per cent of patients showed 20-78% reduction in wound size. In 14% of patients treatment had to be discontinued. All patients showed improvement in granulation tissue and reduction in bacterial isolates and tissue oedema.

**Conclusions:**

The application of subatmospheric pressure or negative pressure promotes healing in a wide range of clinical settings and is an advanced wound healing therapy that can optimize patient care, promote rapid wound healing and help manage costs. It may be used in most instances in both hospital and community settings.

## INTRODUCTION

The use of subatmospheric pressure for wound healing was first investigated by Argenta *et al.*,[1] as a means of promoting wound healing and studies were later translated into clinical settings with numerous authors reporting the advantages of using subatmospheric pressure for wound healing.[2–20] The initial device consisted of a foam piece, embedded plastic tubing and a suction machine. The foam was placed over the wound and the wound was covered with an adherent, occlusive dressing; thus converting the open wound to a controlled closed wound whereby vacuum was created through the plastic tubing by a suction machine and the whole system was developed into what is now established as vacuum-assisted closure (VAC). This paper reviews the use of VAC over a five-year period from January 2000 to January 2005 at the Al Babtain centre for burns and plastic surgery, Kuwait.

## MATERIAL AND METHODS

Between January 2000 and January 2005 a series of 50 consecutive patients at the Al-Babtain centre for burns and plastic surgery, Kuwait were subjected to VAC therapy. All patients were explained the choice of available treatment options and consented to undergo VAC therapy. Patients were classified by diagnosis: Group 1: pressure sore - sacral (n= 3), trochanteric (n=6), ischial (n= 2); Group 2: ulcers (n =11); Group 3: traumatic soft tissue wounds (n =15); Group 4: extensive tissue loss from the abdominal wall perineum, thigh and axilla (n =5); Group 5: sternal dehiscence wounds (n =4) and Group 6: wounds from flap necrosis (n =4). The technique of VAC application used in all patients was similar to that described by Argental *et al*.[1]

**Figure 5:**
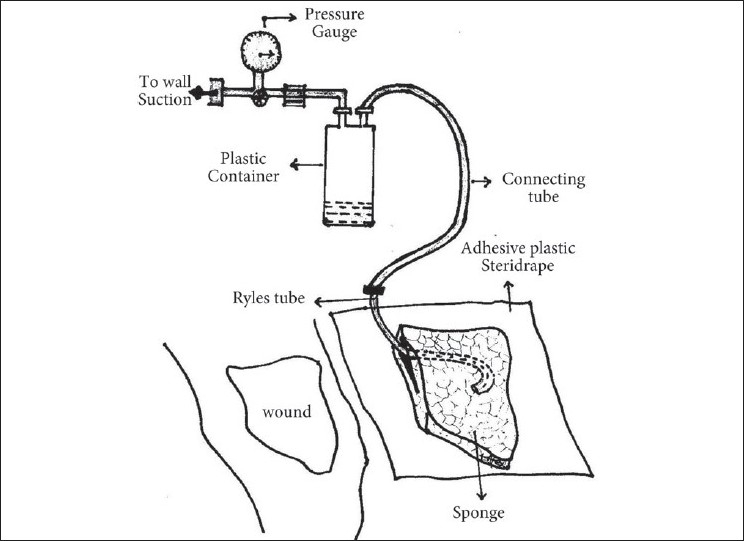
Line diagram of locally assembled VAC apparatus using wall suction unit

In the majority of patients (n=32), vacuum pressure (125 mm of Hg) was applied using a wall suction unit. Figure 5 shows a line diagram of VAC using the wall suction available in all hospital wards. This is connected to a pressure gauge to adjust the pressure which in turn is connected to an outlet of a canister which pools in all the wound secretions. The inlet of the plastic canister in turn is connected to a perforated tube (Ryle's tube), the end of which is buried into a sponge piece cut in the shape of the wound. The tubing along with the sponge is then covered by an adhesive steridrape that is stuck on the skin to make an airtight seal. The pressure is adjusted to 125 mm of mercury and the VAC therapy is started. In the remaining patients, a portable VAC machine was used. While continuous pressure was used in the wall unit, intermittent pressure was applied with the portable VAC machine. All patients were photographed, wound sizes were documented and wound swab cultures were taken prior to the VAC application, during each week of therapy and on termination of VAC treatment. Wounds in this study were treated until they healed completely, until they reduced in size or improved in nature so that a simpler surgical procedure could be carried out to close the wound or the patients refused further treatment.

## RESULTS

A total of 50 patients were subjected to VAC therapy. Of these 33 were males and the rest females. The age of the patients ranged from 5-72 years. In eight patients VAC therapy resulted in complete healing and closure of the wounds. Seven patients refused to continue with the treatment. Further, all wounds were less exudative following VAC therapy. Four patients refused treatment because the wall unit restricted their mobility significantly. Two patients had pain on VAC application while in one patient severe allergy resulted in the surrounding skin from the adherent occlusive dressing warranting termination of VAC therapy. In the rest of the 35 patients, the wounds showed reduction in sizes ranging from 20-78%. All patients showed some improvement in the quality of granulation tissue. Microrganisms isolated from the wound prior to VAC therapy (n=40) included *Staphylococcus aureus, Streptococcus, Klebsiella, Proteus, Pseudomonas,* Methicillin-resistant *staphylococcus aureus* and *Acinobacter*. These organisms were significantly less isolated on termination of therapy. In 40 patients micro-organisms were isolated prior to VAC therapy while in post VAC, they were isolated only in 22 (n=20). The time period of VAC application ranged from five days to four weeks while the healing period of the wounds ranged from two to six weeks.

### Representative cases

#### Patient 1

A 55-year-old gentleman was admitted with a post-traumatic wound of the heel along with osteomyelitis of the calcaneum. Figure 1a shows the picture of the heel wound following thorough debridement. The wound was subjected to VAC therapy using a wall suction unit at a continuous pressure of 125 mm of Hg for three weeks. Figure 1b shows the heel wound following VAC therapy resulting in a three-dimensional reduction in the wound size along with coverage of the exposed calcaneum with granulation tissue. The wound was then covered by a split-thickness skin graft and it healed completely.

**Figure 1a:**
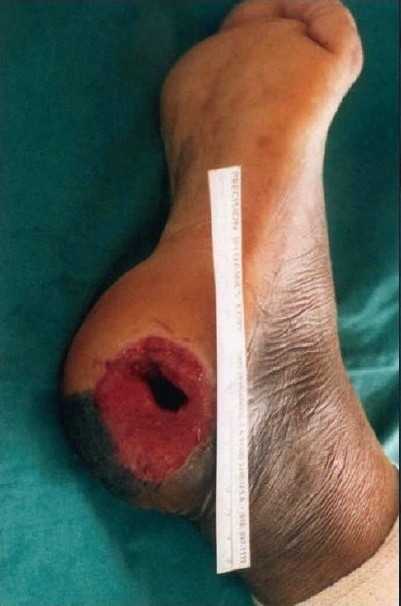
Photograph of the heel showing a post-traumatic infected wound with osteomyelitis of calcaneous bone following debridement

**Figure 1b:**
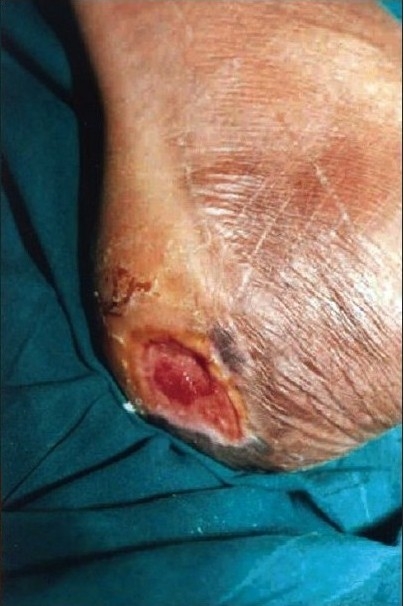
Photograph showing a contracted heel wound covered by granulation tissue following three weeks of VAC therapy

#### Patient 2

A 43-year-old diabetic male, on insulin for the past 10 years, was referred with an abscess wound over the dorsolateral aspect of the right foot [Figure 2a]. The wound was debrided and subjected to VAC therapy using a wall suction unit at a continuous pressure of 125 mm of Hg. Figure 2b shows a reduction in the wound size along with an increase in granulation tissue. Further debridement was carried out, following which VAC therapy was restarted. The wound contraction continued and the granulation tissue improved following which a split-thickness skin graft was applied. Figure 2c shows a completely healed diabetic wound. The diabetes was managed by subcutaneous insulin therapy based on eight-hourly blood sugar level estimation.

**Figure 2a:**
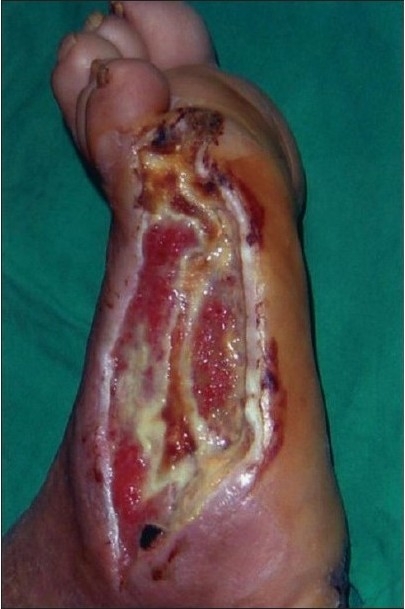
Photograph showing a post-diabetic abscess of the right foot prior to the VAC application

**Figure 2b:**
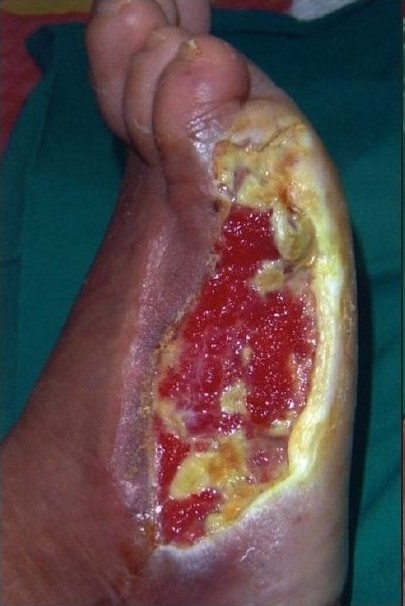
Photograph showing increase in granulation tissue following three weeks of VAC application

**Figure 2c:**
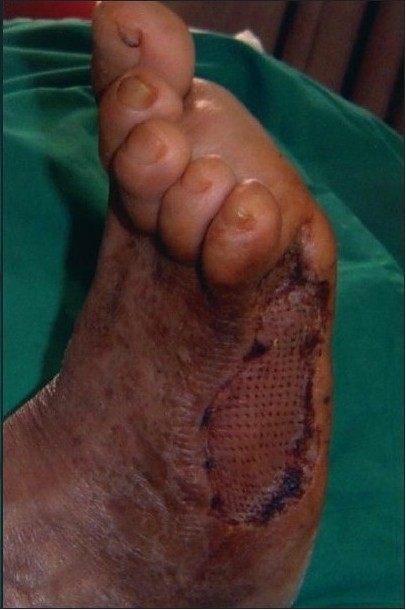
Photograph showing completely healed and contracted wound following six weeks of VAC application and split-thickness skin grafting

#### Patient 3

A 12-year-old boy was referred with history of blast injury to the right lower limb. The patient had a fracture of the tibia and fibula with vascular compromise. The fracture was fixed by external fixation following radical debridement and referred for soft tissue coverage. On examination, there was a defect of 10×12 cm with tissue loss and tibialis anterior tendon lay exposed along with the fracture site [Figure 3a]. Though the vessels were compromised the limb was viable and in view of the traumatized soft tissue around the defect site it was decided to put the wound on VAC therapy [Figure 3b]. Following three weeks of VAC therapy the wound improved remarkably [Figure 3c] and was covered by a split-thickness skin graft subsequently [Figure 3d]. The underlying tibial fracture healed on the same external fixators.

**Figure 3a:**
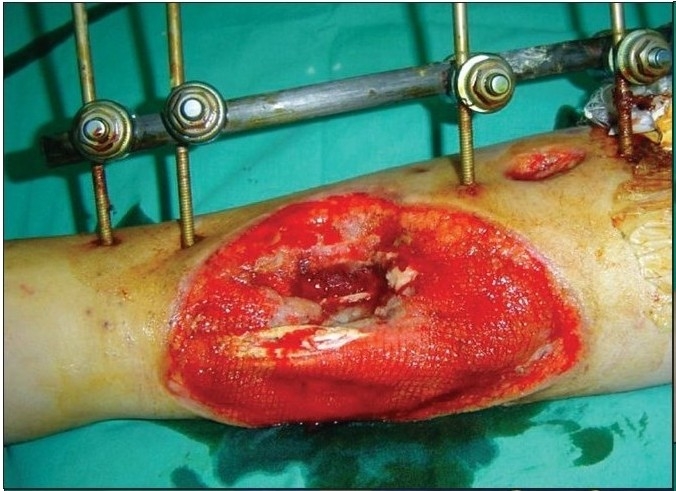
Photograph showing right leg middle third wound with exposed fractured tibia and bare tendons stabilized by external fixators

**Figure 3b:**
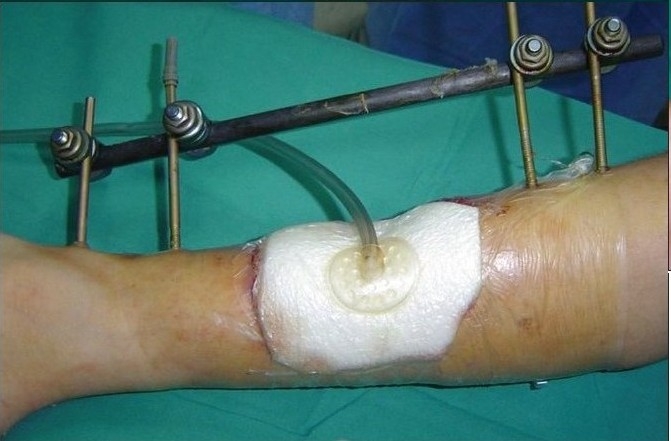
Portable VAC applied on the wound

**Figure 3c:**
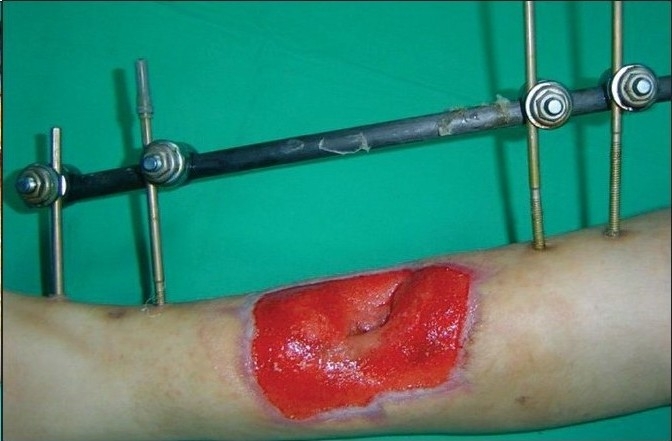
Three weeks following VAC application the bare bone and tendon has been completely covered by granulation tissue

**Figure 3d:**
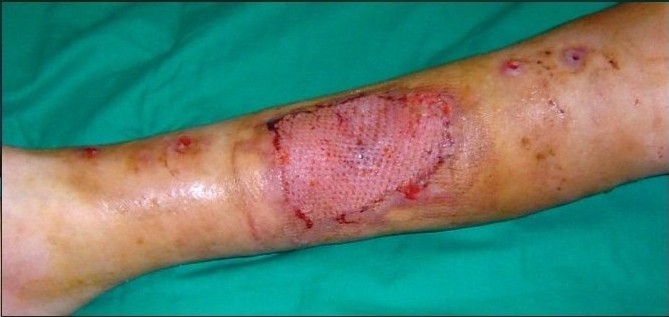
Photograph showing a healed wound following split-thickness skin graft

#### Patient 4

A 42-year-old male was referred following a road traffic accident to the right lower limb. The patient sustained a fracture of the right ankle joint which was managed by external fixation and debridement of the medial ankle wound had been done. He had a 5×7 cm ankle wound with slough and exposed lower end of tibia and ankle joint [Figure 4a]. The wound was put on VAC therapy for two weeks whereby the slough disappeared and was replaced by granulation tissue which covered the exposed tibia and ankle joint [Figures 4b and c] over a period of one to three weeks. The wound was subsequently skin grafted [Figure 4d]. The patient recovered completely and was able to return to his normal work routine.

**Figure 4a:**
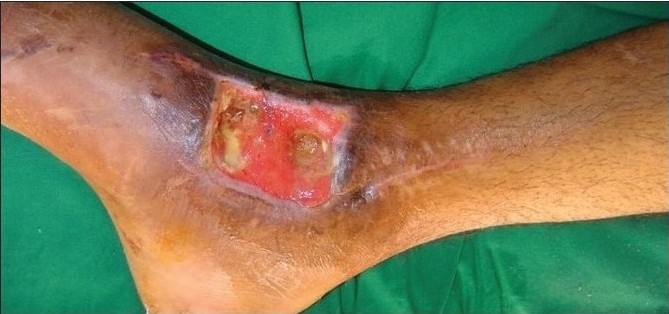
Photograph showing ankle wound with exposed ankle joint and tendons

**Figure 4b:**
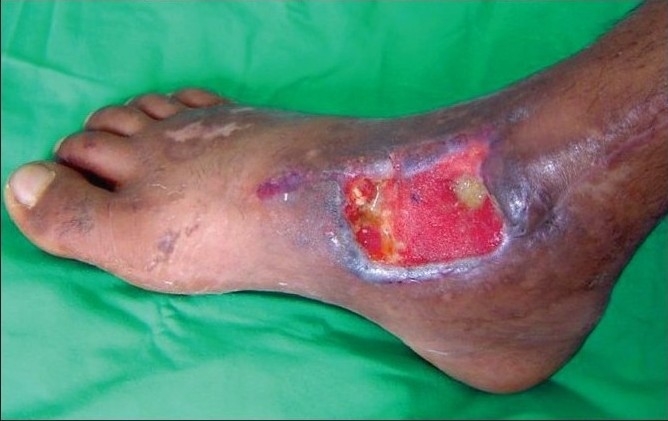
Photograph showing ankle wound one week after VAC therapy

**Fig 4c:**
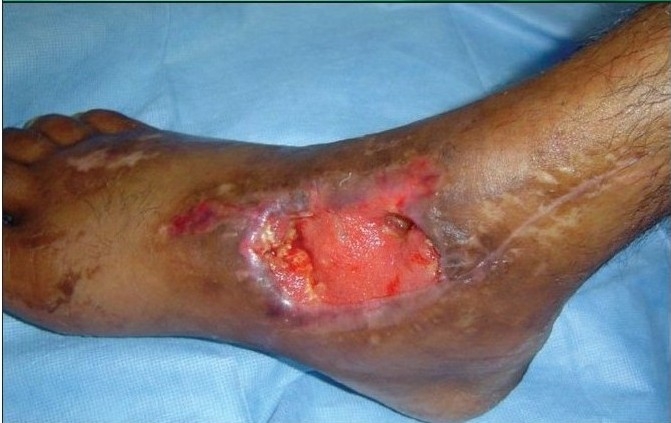
Photograph showing well-granulated wound after three weeks of VAC application

**Figure 4d:**
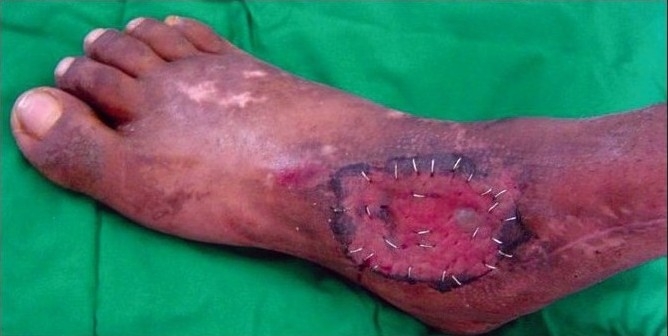
Photograph showing healed wound following split-thickness skin graft

## DISCUSSION

Wound healing is the culmination of a complex interaction between the reticuloendothelial and immune systems, with a number of internal and external factors influencing the outcome. In the past, a number of adjuvant therapies like hyperbaric oxygen, growth factors and skin substitutes have been used to enhance wound healing. Over the past few years, the VAC has become a major player with a number of surgical specialities including it in their armamentarium for wound healing. VAC referred to as vacuum-assisted fascial closure (VAFC) has been used in the management of open abdomen, especially where gross contamination, massive bowel oedema and continued bleeding at re-exploration may be present. The VAC system can be an effective and economically viable method of containing fistula effluent, protecting the skin of patients with enterocutaneous fistulae and promoting healing of the fistula.[2,3] VAC is of great help in the treatment for post-sternotomy mediastinitis leading to excellent survival and a very low failure rate when compared with conventional treatment by surgical revision, closed irrigation, or reconstruction with omentum or pectoral muscle flaps.[4] VAC further improves early postoperative lung function and reduces pulmonary complications in patients with large sternal wounds.[5] Vacuum-assisted closure devices may improve the viability of split-thickness skin grafts during vulvo-vaginal reconstruction where infection and sloughing of the grafts may occur, especially in irradiated sites.[6,7] In the treatment of patients with malignant lesions, VAC coverage allowed repeated brachytherapy and external-beam applications.[8] Sequential irradiation had no effect on neighbouring flap tissues, which healed without impairment following transposition. Thus, the combination of VAC and brachytherapy can effectively replace circumstantial and laborious intraoperative radiotherapy procedures.

VAC has been effectively applied to heal or improve wounds in a variety of circumstances: exposed bone in burn patients, exposed tendons, extensive wounds following necrotizing fasciitis, salvage of exposed alloplastic materials in irradiated wounds, exposed instrumentation in spinal wounds, calciphylaxis-induced chronic wounds, improving bioengineered tissue grafts in diabetic foot wounds, in the management of infected vascular grafts in vascular surgery and in chronic pilonidal sinuses.[9–16] It has also been used in intraoral as well as orbital reconstruction.[17,18] The use of VAC has been carried out in all age groups including premature neonates with extraordinary soft tissue defects to assist in closing wounds.[19] Vacuum-assisted healing has also been used in protocol management of cases where two-stage operations have been planned to close the wound completely or to prepare the wound for the second stage allowing elective planning of the definitive reconstructive surgery without jeopardizing the wound or its outcome.[20–22] The army too has incorporated VAC in its use of new therapeutic approaches to treat soldiers injured in war.[23]

*In vitro* studies of VAC therapy have demonstrated fibroblast migration, increased proliferation through mitosis and the creation of a micro-environment that promotes granulation tissue formation.[24–30]

In an experimental study in rabbits, it was found that VAC increased capillary calibre and blood volume, stimulating angiogenesis thereby improving the blood circulation in wounds. Further, it narrowed endothelial spaces and restored the integrity of capillary basement membranes causing a decrease in the permeability of blood vessels and wound oedema by removing excessive fluid from the wound bed.[24] Significantly, an increased rate of granulation tissue formation was also shown to occur with both continuous and intermittent VAC application.

The bacterial colonization of a wound is a recognized detrimental factor in the multifactorial process of wound healing. While the harmful effects on wound healing are recognized to correspond to a level of > 10^5^ colonies of bacteria per gram of tissue, studies have found that there is no consistent effect of bacterial clearance with the application of VAC in wound management. While some studies have found tissue bacterial counts to have significantly decreased after four days of application, other studies have shown that bacterial colonization increased significantly with wound VAC therapy and remained in a range of 10^4^ −10^6^.[25,26] Non-fermentative gram-negative bacilli showed a significant decrease in VAC-treated wounds, whereas *Staphylococcus aureus* showed a significant increase in VAC-treated wounds. Despite this finding, the beneficial effects of this treatment modality on wound healing were noted in most cases.[27]

Wound surface area reduction was significantly larger in VAC-treated wounds compared to conventionally managed wounds.[28] While close to the wound edge relative hypoperfusion was observed in muscular tissue, the distance from the wound edge to the position at which the blood flow was increased was shorter than that in subcutaneous tissue. The hypoperfused zone was larger at high negative pressures and was especially prominent in subcutaneous tissue. Wound fluid partial pressure of oxygen and lactate levels were increased after 60 min of VAC therapy: this combination is known to promote wound healing which may be the result of changes in the microvascular blood flow. It may be beneficial to tailor the negative pressure used for VAC therapy according to the wound tissue composition.[28] When VAC therapy was terminated, blood flow increased multifold, which may be due to reactive hyperemia. A low negative pressure during treatment may be beneficial, especially in soft tissue, to minimize possible ischemic effects and intermittent VAC therapy may further increase blood flow.[29] *In vitro* studies have revealed that cells allowed to stretch tend to divide and proliferate in the presence of soluble mitogens, whereas retracted cells remain quiescent. In a computer model (finite element) of a wound, which simulated VAC application it was shown that most elements stretched by VAC application experienced deformations of 5-20% strain, similar to *in vitro* strain levels shown to promote cellular proliferation. The application of micromechanical forces through VAC may be a useful method with which to stimulate wound healing through promotion of cell division, angiogenesis, and local elaboration of growth factors.[30] The cyclical application of subatmospheric pressure alters the cytoskeleton of the cells in the wound bed, triggering a cascade of intracellular signals that increases the rate of cell division and subsequent formation of granulation tissue.[31] The application of the VAC technique during wound healing increases the expression of the apoptotic modulation-related protein Bcl-2 and affects the expression of NGF/NGFmRNA, which may promote the wound healing process.[32] VAC can promote healing of chronic wounds through depressing the expressions of MMP-1, 2, 13 mRNA and protein synthesis, depressing the degradations of collagen and gelatine.[33] Studies of VAC therapy on blood vessels in pigs for 12 h have shown to enhance an endothelin Type A and Type B receptor-mediated vasoconstriction. This may be compensated for by a more efficacious endothelium-dependent vasodilatation. No spontaneous bleeding, perforation, dissection, or other macroscopic change could be observed in the arteries exposed to VAC therapy.[34] Subatmospheric pressure created in the wound promotes wound healing through secondary or tertiary intention by preparing the wound bed for closure, reducing oedema, promoting granulation tissue formation, enhancing tissue perfusion and by removing exudates and infectious material. Further the application of a macro strain (centripetal force) draws the wound edges together while a micro strain (forces on the micro circulation) causes cell stretching, increased tissue perfusion, cell mitosis and fibroblast proliferation, thus creating an environment of wound healing at the cellular level.

While both continuous suction and alternating pressure cycles have been used, the optimal subatmospheric pressure for wound, healing appears to be approximately 125 mm Hg utilizing an alternating pressure cycle of 5 min of suction followed by 2 min off suction.[31] In most of our cases, the normal wall suction unit was used. However, one has to continuously monitor the vacuum, while at the same time the patient is hospital-bound and has to be managed as an inpatient.

In patients with diabetes mellitus, presence of a high wound bacterial load and complications such as polyneuropathy and peripheral angiopathy lead to foot ulcers, gangrene, and osteoarthropathy which may necessitate minor or major amputation. Free tissue transfer or local flap surgery is often difficult or even impossible owing to an impaired arterial circulation. The VAC can be of great benefit in such cases by helping to heal the wounds satisfactorily. Because it is a closed system of treatment, it has the added benefit of minimizing exposure of staff and other patients to communicable diseases.

Pain was not a predominant feature although some patients complained of discomfort during initial application of VAC; reduction in suction pressure helps to minimize it. In all our patients where VAC therapy was used, wound care was simplified and healing accelerated. The VAC device allowed optimal wound closure by preparing the wound for skin grafting or flap closure when required, in cases where the wounds did not heal completely with VAC therapy. Its use in the perineum presents a challenge, but with proper application, even the most complex perineal wounds can be healed. A cost-benefit analysis revealed that VAC therapy was genuinely cost-effective.[35] VAC wound care is particularly beneficial in children as there are less frequent dressing changes, and outpatient management, resumption of daily activities including returning to school, and a high degree of child tolerance is seen.[36]

The VAC should be used with caution over exposed vessels as it has been shown to cause erosion and haemorrhage of the left anterior tibial artery.[37] Application of VAC may be difficult in extensive wounds which lack an intact skin surface and newer and better techniques need to be developed for such patients. In one study retained sponge and device malfunction were observed in some patients.[36] Toxic shock syndrome has been reported as one of the unusual complications following VAC application.[38] Absolute contraindications[39] for use of VAC therapy include direct contact of the sponge with exposed blood vessels, anastomotic sites, organs and nerves. Also, application over wounds with malignancy and non-enteric and unexplained fistulas should be avoided. Relative contraindications[39] include VAC application in wounds that are likely to bleed, infected or friable blood vessels in wound vicinity, wounds with bulky slough and necrotic tissue, patients without adequate wound haemostasis and patients on anticoagulants or platelet aggregation inhibitors. These patients should be monitored for sudden and serious active bleeding which can be fatal if untreated.

The VAC device is an extremely versatile tool in the armamentarium of wound healing. Application of controlled subatmospheric pressure creates an environment that promotes wound healing. In order to heal a chronic wound, a number of factors need to be addressed like oxygen delivery to the tissue, wound nutritional requirements, a moist environment, debridement of slough and necrotic tissue and correction of associated medical illnesses. While in some patients these factors may be easily dealt with, in others adjunctive therapies like hyperbaric oxygen, growth factors and skin substitutes can help to heal the wounds. The VAC is an additional tool that can be of great benefit in healing wounds in selected patients. If utilized selectively and judiciously, VAC is a valuable tool; but its prolonged application over several weeks at the expense of early surgical reconstruction needs to be deplored. Ideologically, the VAC was never meant to replace surgical interventions, only to complement them. VAC is generally well tolerated and with few contraindications or complications, is fast becoming a mainstay of current wound care.
